# Effects of urban association on the movement ecology of hamadryas baboons (*Papio hamadryas*) in Saudi Arabia

**DOI:** 10.1371/journal.pone.0354133

**Published:** 2026-07-23

**Authors:** Zaffar Rais Mir, Paula Pebsworth, Jack Hardman, Saleh Ala’amri, Ali Alahmari, Khalid Almalki, Ahmed Boug

**Affiliations:** 1 National Center for Wildlife, Riyadh, Saudi Arabia; 2 The University of Texas, San Antonio, Texas, United States of America; 3 Newcastle University, Tyne and Wear, United Kingdom; Tshwane University of Technology, SOUTH AFRICA

## Abstract

Human–wildlife conflict involving primates is an emerging conservation concern in rapidly urbanizing regions. The hamadryas baboon (*Papio hamadryas*), the only native non-human primate of the Arabian Peninsula, increasingly exploits anthropogenic food sources, intensifying human–baboon conflict across southwestern Saudi Arabia. To examine how human-modified environments influence baboon spatial ecology; we fitted GPS collars to ten adult males representing groups that are regarded as living in urban, natural and semi-natural environments across five regions. Over nine months, we collected 13,962 location fixes and analyzed them using empirical variograms, home range analysis, and minimum daily path length estimates. Variogram analyses indicated that baboons living in a natural environment exhibited the greatest space-use extent, semi-natural individuals showed intermediate and more variable space-use dynamics, whereas baboons living in urban areas displayed restricted movement. Hamadryas baboons living in natural environments exhibited the largest home ranges with a median of 8.79 km^2^ (range: 6.19–13.86 km^2^) and minimum daily path lengths with median 3.33 km (range 0.02–20.6 km), whereas baboons living in urban areas maintained the smallest ranges with a median of 1.31 km^2^ (range: 1.24–1.79 km^2^) and the minimum daily path lengths with a median of 2.16 km (range: 0.01–4.85 km). The proportion of daytime spent in urban areas increased along the gradient, 7.2% (natural), 40.6% (semi-natural), and 55.0% (urban). These findings support the Resource Dispersion Hypothesis, suggesting that food predictability shapes baboon space use more than resource scarcity. The study strengthens ongoing conflict-mitigation efforts by providing insights into baboon movement patterns and supporting the ecological differentiation of natural, semi-natural, and urban baboon groups, thereby informing category-specific management interventions.

## Introduction

Human–wildlife conflict is a growing conservation concern worldwide, and primates are often central to these conflicts due to their adaptability [1,2]. Species from the genera *Macaca*, *Papio*, and *Chlorocebus* are frequently reported in conflict with humans [3–6]. Their social organization, cognitive abilities, opportunistic feeding, effective communication, and agility make them particularly difficult for people to manage [7]. Among them, baboons (*Papio* spp.), like macaques, are considered as highly intelligent and behaviourally flexible primates, allowing them to exploit human-dominated landscapes like cultivated areas, refuse sites, and residential areas [2,6,8,9]. Such conflicts not only threaten human livelihoods but also foster overall negative attitudes toward wildlife [10], often resulting in retaliatory actions such as chasing, injuring, or killing baboons, which can disrupt their social structure, alter ranging patterns, and increase stress-related behaviors [8,11–13].

The hamadryas baboon (*Papio hamadryas*), the only non-human primate species native to the Arabian Peninsula, provides a particularly relevant case [6,14]. In Saudi Arabia, hamadryas baboons occupy the Sarawat and Hijaz Mountain ranges along the Red Sea coast, where their distribution is primarily influenced by rainfall patterns, temperature, vegetation cover, and the availability of steep cliffs for sleeping [14,15]. In areas lacking cliffs, they have adapted to using trees as alternative sleeping sites [14]. Once considered relatively few in number, recent surveys indicate that the non-urban baboon population has increased from approximately 1,079 individuals in 1989 to nearly 19,500 individuals in 2025 [14]. In parallel, urban associated baboons have also become increasingly prominent, with recent estimates indicating more than 24,000 individuals associated with human-modified environments [14]. This increase is closely tied to the rapid human population growth and urban development in southwestern Saudi Arabia resulting in new foraging opportunities through food provisioning and increased availability of food wastes [14]. The decline in predator populations is also considered as an important factor [16]. Over recent decades, hamadryas baboons in Saudi Arabia have shown remarkable flexibility in adapting to anthropogenic environments. Around human habitations baboon troops rely heavily on waste facilities, roadside dumps, farms and household garbage, with some urban-associated troops exceeding 500 individuals. While Biquand *et al*. [17] documented early forms of this urbanization of baboons, recent surveys confirm that conflicts have escalated sharply [6,18]. In a countrywide study covering six administrative regions, 63% of interviewed residents reported daily incursions by baboons, with 60% experiencing crop or property damage—losses ranging from US$250 to over US$1,300 per household annually [6]. Complaints to authorities highlight not only crop destruction but also house incursions, attacks on children, and livestock losses. Such pressures have fueled widespread hostility, with 67% of respondents expressing negative attitudes toward baboons [6]. Overall, the shift from predominantly natural foraging to reliance on anthropogenic food has intensified human-baboon conflict in Saudi Arabia, making coexistence an urgent conservation and socioecological challenge.

Given the severity of the problem, spatial ecology provides a critical, evidence-based framework for conflict mitigation. By quantifying how baboons use natural and urban landscapes and visualizing their spatial patterns, researchers can pinpoint hotspots of human–baboon interaction, identify core foraging areas and movement corridors, and determine the landscape features driving conflict [1]. This is also theoretically important because, under the Resource Dispersion Hypothesis, the distribution, abundance, and predictability of food resources are expected to shape ranging patterns and home-range size. These insights are essential for developing targeted strategies to improve human-baboon coexistence. The ecological knowledge generated can guide development of locally appropriate conservation policies that address stakeholder concerns while maintaining viable baboon populations; policies based on rigorous spatial data are far more likely to be practical and accepted [19]. For example, in the Cape Peninsula, Hoffman & O’Riain found that troops whose sleeping-sites were closer to the urban edge showed higher levels of human–baboon conflict [1]. They recommend that management should intervene and discourage the use of sleeping-sites near the urban edge in favour of those further away. Thus, integrating GPS-derived maps of home ranges, space use and seasonal movements into management planning enables targeted interventions (e.g., waste management, buffer zones, seasonal patrols, and community engagement) that directly reduce encounters and support coexistence [20]. Studies from sub-Saharan Africa and other regions have documented significant variation in baboon ranging behavior in response to habitat type, anthropogenic food availability, and seasonal climatic variation [19,21–25]. However, comparable data from the Arabian Peninsula are scarce, particularly regarding how baboons in this region respond to the growing human development and increasing intra-specific competition.

Recent technological advances have greatly improved our ability to collect such spatial data and address this gap. GPS telemetry collars allow researchers to record animal locations at fine temporal scales over several months. Henriquez *et al.* deployed GPS collars on hamadryas troops in Ethiopia and found that traditional estimates had greatly underestimated their home-range sizes; the collars also revealed previously unknown sleeping sites and seasonal range shifts [20]. Similarly, lightweight GPS collars (e.g., Televilt “Tellus”, Advanced Telemetry Systems models) had been used on Guinea baboons (*Papio papio*) and chacma baboons (*Papio ursinus*) to gather multi-season movement data [20,26–28]. These biologging tools provide high fix success rates and 3D accuracy, enabling detailed analyses of daily travel paths and space use data that are especially useful in conflict studies [29].

Data on baboons in Saudi Arabia obtained through these technologies are, however, still limited. The only previous movement study, conducted on a single baboon troop in 1995 using VHF collars, provided valuable baseline information but lacked the spatial and temporal resolution required to understand how baboons respond to contemporary patterns of urbanization [30]. Addressing this gap is essential for interpreting behavioral adjustments to shifting resource landscapes and for informing effective conflict-mitigation measures.

Guided by the Resource Dispersion Hypothesis (RDH) [31,32], which posits that ranging behavior is shaped primarily by the dispersion and predictability of resource patches rather than by their absolute abundance, we investigated how hamadryas baboon troops differed in their space-use strategies along a gradient of human association. For this purpose, we classified baboon troops as natural, semi-natural, or urban based on field observations, behavioral context, and the degree of reliance on human-modified habitats and anthropogenic resources, following previous classifications [14,15]. The RDH framework has been applied to primates, including baboons, where access to spatially concentrated and predictable food sources, particularly those associated with human activities, has been shown to influence ranging patterns, group cohesion, and movement behavior [33]. We aimed to quantify variation in home-range size, minimum daily path lengths, and temporal range stability along the behavioral gradient. This study provides an ecological context for interpreting differences among baboon troops that vary in their degree of human association, enabling targeted management interventions. Consistent with RDH, we hypothesized that baboons relying on patchily distributed natural resources would exhibit larger and more variable ranges, whereas those living in urban areas (exploiting clumped and predictable anthropogenic foods) would show reduced movement and smaller home ranges.

## Methods

### Study area

**Fig 1:**
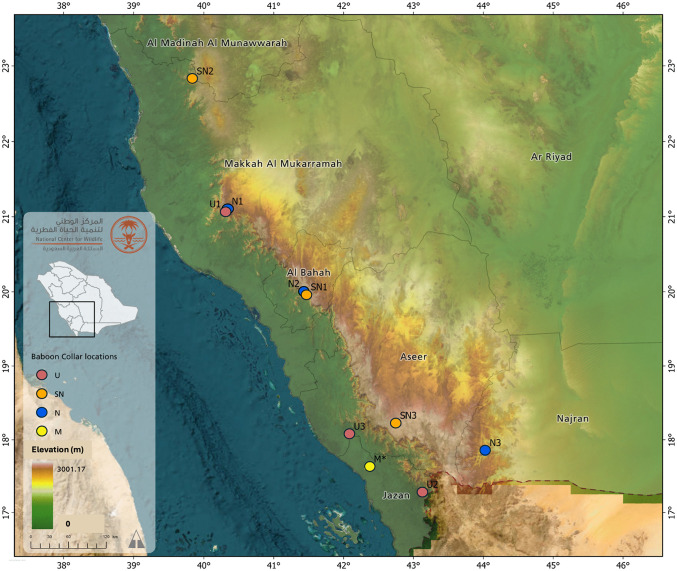
GPS-collared hamadryas baboon (*Papio hamadryas*) locations across south-western Saudi Arabia. Colors indicate behavioral categories: urban (U, red), semi-natural (SN, orange), natural (N, blue), and a malfunctioned collar (M, yellow). Elevation ranges from 0 to 3001 m (green to yellow gradient). The inset shows the study area within Saudi Arabia. (Basemap: Esri. Data sources: Esri, Maxar, Earthstar Geographics, and GIS user community.).

We conducted this GPS collaring study at ten locations across five regions spanning the distribution range of the hamadryas baboons, which occurs naturally in six administrative regions of Saudi Arabia: Al Madinah Al Munawwarah, Makkah Al Mukarramah (Makkah), Aseer, Al-Bahah, Jazan, and Najran (Fig 1) [14,15]. In Saudi Arabia, baboons are specifically distributed along the Sarwat and Hijaz mountain ranges (Fig 1). Sarwat mountain range is situated in the southwestern region of the country. The Hijaz Mountain Range stretches along the western region, running parallel to the Red Sea coast. Baboon distribution range encompasses a diverse range of altitudinal gradients with mountains spanning in altitudes up to 3000 m above sea level, supporting distinct habitats characterized by varying climatic conditions [34]. General ecological conditions of the area mirror those in the mountain ranges of Eritrea and Ethiopia, where hamadryas baboons also occur [35]. Valleys characterize the landscape, providing vital water sources and facilitating the growth of hardy shrubs, grasses, and perennial plants [36]. High-altitude plateaus create cooler alpine environments with distinct floral diversity [34,36]. The mountain flora exhibits diverse adaptive strategies that enable persistence across a wide range of climatic conditions [36]. The lower altitudes are home to drought-tolerant species, such as acacias *(Vachellia* spp.) and other xerophytic plants, while higher elevations feature species such as junipers (*Juniperus phoenicea* and *Juniperus procera*) and native olives (*Olea europaea*). The fauna of the area is diverse, including a range of mammals, birds, reptiles, and invertebrates [15,37–40].

### Data collection

To examine baboon movement, we selected ten different troops exhibiting various levels of human interaction across five regions of Saudi Arabia and deployed a GPS collar on one adult male from each troop in August 2022. Data collection continued until May 2023, when the collars’ batteries were depleted, although the study was ideally designed to obtain at least one full year of tracking data. One of the ten collars malfunctioned after six days; therefore, we excluded data from this collar from the analyses. We designed the sampling to ensure broad spatial coverage of hamadryas baboon populations across southwestern Saudi Arabia and to represent different behavioral categories based on their level of interaction with humans. Accordingly, the sample included two troops each from Makkah, Al Bahah, and Jazan; three from Aseer and one from Najran, where baboons occupy only a limited area. The collaring sites for the study troops were located at least 10 km apart to ensure spatial independence among the sampled groups. To further ensure that the sampled groups were different, troop identity was confirmed through simultaneous collaring, field observations, and information from local residents and rangers. In addition, GPS locations and estimated home ranges did not overlap during the study period, indicating that the collared animals belonged to separate troops. We used Tellus Light GPS collars (Followit AB, Lindesberg, Sweden), each weighing less than 240 g. These collars utilize the Iridium satellite system, which features two-way communication between the collar and a computer server, and has an automatic self-releasing mechanism so that the collar can be removed at any time. The collars provide the date, time, latitude/longitude, altitude, 2D/3D, number of satellites, H-DOP, TTF (time to fix), mortality notification, and battery voltage. To conserve battery life, we programmed the collars to record eight GPS locations per day (at 0000, 0300, 0600, 0900, 1200, 1500, 1800, and 2100 hours). We disabled the VHF beacon, set the collars to transmit data after obtaining 11 locations (the maximum setting), and enabled remote drop-off for collar recovery. We labelled the collars as N1–N3 (natural groups), SN1–SN3 (semi-natural groups), and U1–U3 (urban groups).

Certified veterinarians trapped, sedated, and took morphological measurements from each potential GPS collar candidate. We deployed GPS collars only on healthy adult male baboons for which the collar mass was less than 5% of body mass. Males were specifically selected to gain insights into group movement patterns. In the social system of the hamadryas baboon, adult males typically constitute the social core of the group and coordinate movements of their associated individuals; therefore, they serve as reliable representatives of group spatial behavior [41]. We fitted each collar with an approximately two-finger gap between the collar and the animal’s neck. This degree of tightness allowed the baboon to groom under the collar but not pull it over their heads. After collaring the baboons, we monitored them continuously until they recovered completely from the anesthesia and rejoined their respective troops. Recovery took approximately two hours, after which each individual rejoined its troop. We monitored baboon movements and collar battery levels daily using Followit’s online platform. According to Followit guidelines, the battery life is 3 years and collars should be released once the battery voltage drops below 2.7 V. However, in this study collar U3 was the first to reach this threshold on 18 January 2023, and the remaining collars were depleted by 11 May 2023.

### Data analysis

We processed and analyzed all spatial and movement data obtained from GPS collars in R (version 4.5.2) using the packages ctmm [42], move, and adehabitatHR. Data screening involved removing erroneous GPS fixes with unrealistic step lengths or speeds exceeding known baboon movement capacities (>10 km/h). We projected cleaned datasets into the WGS 84 / UTM Zone 37N coordinate system for consistency in spatial analysis.

We assessed the performance of GPS collars based on the time required to obtain a positional fix (“time to fix”). For each location attempt, the collar recorded the duration between activation and successful satellite acquisition. If no fix was obtained within 89 seconds, we classified the attempt as a “time-out.” To evaluate fix efficiency, we grouped times into three categories: 30–49 s (Category 1), 50–69 s (Category 2), and 70–89 s (Category 3).

To assess spatial autocorrelation and temporal structure in movement behavior, we computed empirical variograms for each collared individual using the variogram function in ‘ctmm’ package. We visually examined variogram patterns to identify characteristic scales of spatial dependency and temporal range stability and interpreted differences among natural, semi-natural, and urban living individuals in terms of semivariance magnitude and temporal range, providing insight into variation in space-use consistency.

Home range estimation was conducted using the Autocorrelated Kernel Density Estimation (AKDE) approach, which accounts for temporal autocorrelation in GPS data and provides statistically robust utilization distributions [43]. Individual movement models were first fitted using maximum likelihood estimation (ctmm.fit), and the best-supported model (e.g., Ornstein–Uhlenbeck Foraging model; OUf) was selected based on the Akaike Information Criterion corrected for small sample size (AICc). The 95% AKDE ranges were extracted for each individual. For comparison with previous studies, Minimum Convex Polygon (MCP) home ranges were also computed using adehabitatHR [44]. Differences in home range sizes between behavioral categories (natural, semi-natural, and urban) were tested using the non-parametric Kruskal–Wallis test, with significance set at *p* < 0.05.

To quantify the degree of urban use, GPS location data were spatially overlaid on a land-use map derived from Sentinel-2 imagery using the sf and raster packages in R (v4.5.2). Urban boundaries were delineated using built-up area polygons, while non-urban areas included natural habitats such as rocky escarpments, wadis, and vegetation patches. For each collared baboon, GPS fixes recorded between 06:00 and 18:00 h were classified as “urban” or “non-urban.” The proportion of daytime locations falling within urban polygons (near built up areas) was calculated to estimate the percentage of daytime spent in urban areas for each individual. These values were then averaged by behavioral category (natural, semi-natural, and urban) and Kruskal-Wallis test was used to assess differences in the extent of urban space use among groups.

Minimum daily path lengths (MDPLs) were derived from successive GPS fixes collected at 3-hour intervals using the ‘move’ package. Step distances were calculated between consecutive locations and aggregated to daily totals to represent average daily travel distances per individual. Descriptive statistics were computed to summarize MDPL variation across individuals. Differences in minimum daily path length among different categories (natural, semi-natural, and urban) were tested using the Kruskal–Wallis test, followed by post-hoc pairwise Wilcoxon rank-sum tests with Bonferroni correction. All analyses and visualizations were performed in program R.

### Ethical statement

All capture, handling, and GPS collaring procedures were conducted by the National Centre for Wildlife (NCW), Saudi Arabia, which is also the regulatory authority responsible for wildlife research permits. The study was carried out in accordance with NCW’s ethical guidelines for the use of wild mammals in research, ensuring minimal disturbance and welfare of the animals. The study was approved by the Scientific committee of NCW.

## Results

Out of 14,458 programmed opportunities, collectively, nine GPS collars captured 13,962 (96.57%) data points and remained active for an average of 256 days before depletion of batteries. Because the conditions varied between locations, so did the collar results.

For the collars, the time required to obtain a GPS location (time to fix) ranged from 30 to 89 seconds (mean = 38.59, SD = 11.22). If location was not captured in 89 seconds, the collar “timed out”. By category, the GPS points were captured in the following categories; category 1 (30–49 seconds) n = 11,775 (84.34%), category 2 (50–69 seconds) n = 1,733 (12.41%), and category 3 (70–89 seconds) n = 454 (3.25%).

**Fig 2:**
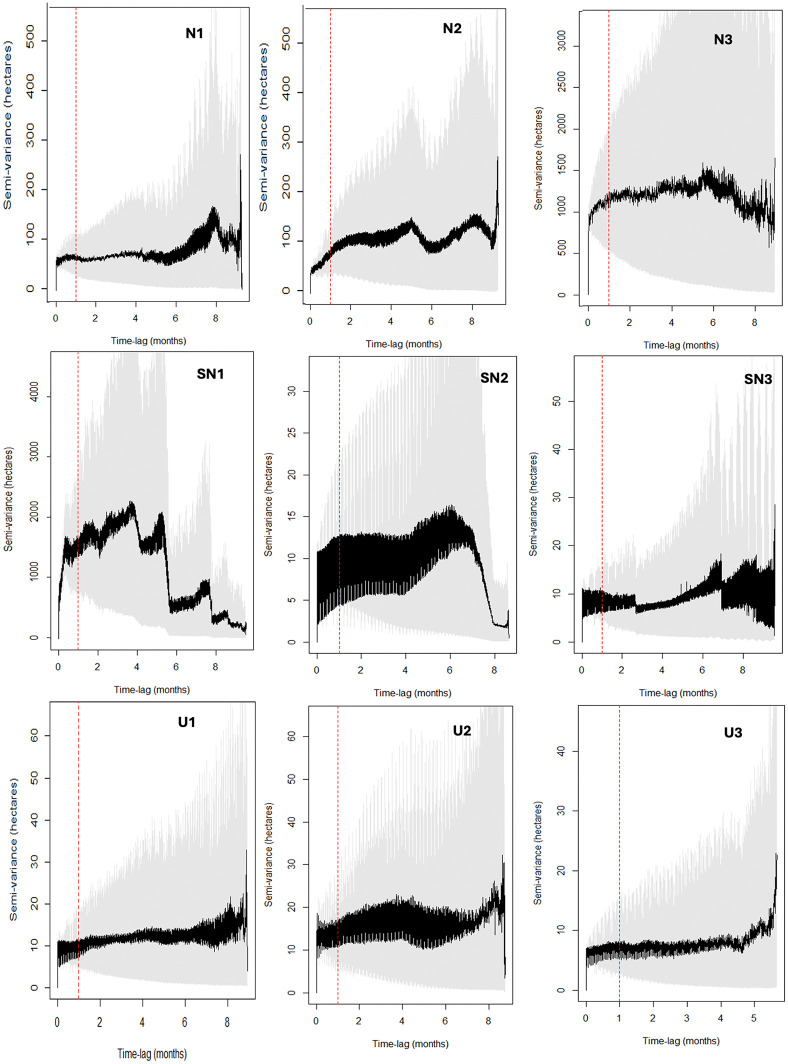
Empirical semivariograms of GPS-collared hamadryas baboons in Saudi Arabia (August 2022-May 2023). The black line shows semivariance over increasing time lag (months). Grey shading represents the model confidence envelope, and the red dashed vertical line marks a one-month reference lag used to facilitate visual comparison. Note that semivariance values differ substantially among individuals; therefore, panels are shown with individual-specific y-axis scales to preserve within-individual variogram structure.

The empirical variograms of baboon movement across different individuals show distinct spatial autocorrelation patterns that align with their classification into natural, semi-natural, and urban groups (Fig 2). Baboons living in natural environments generally showed increasing semivariance across time lags, indicating broader use of space over increasing time lag and extensive ranging behavior, although the highest semivariance values were observed in one individual living in semi-natural environment (SN1). Semi-natural individuals exhibited the greatest heterogeneity in variogram structure with some showing high and temporally variable semivariance, indicating flexible space-use dynamics. In contrast, urban individuals consistently displayed the lowest semivariance values with relatively shallow increases across time lags, reflecting restricted movement and smaller spatial ranges. These differences highlight clear contrasts in movement variability across behavioral categories.

### Home range estimates

**Fig 3:**
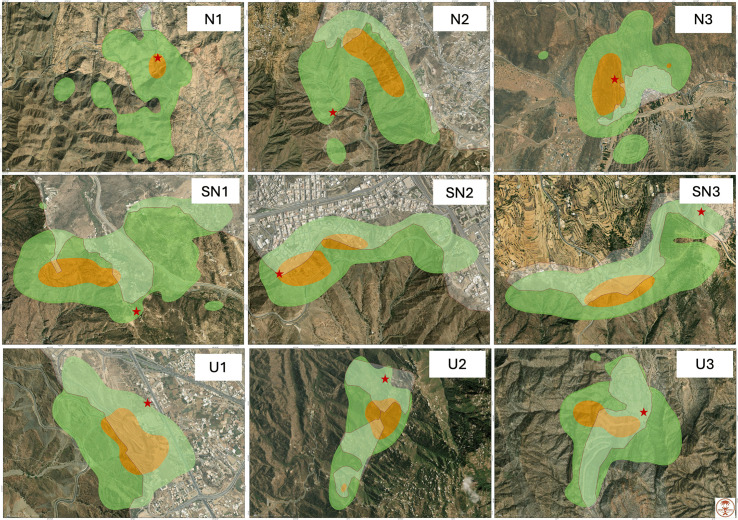
Home ranges of nine GPS-collared hamadryas baboons (IDs U1-U3, SN1-SN3, N1-N3) representing urban, semi-natural, and natural baboon troops in Saudi Arabia (August 2022-May 2023). Home ranges were estimated using the Autocorrelated Kernel Density Estimate (AKDE) with an Ornstein-Uhlenbeck foraging movement model (OUf), showing the 95% (home range) and 50% (core range) utilization distributions. Built-up area polygons used to delineate urban boundaries are shown in semi-transparent light grey, and red stars indicate collaring locations. (Basemap: Esri. Data sources: Esri, Maxar, Earthstar Geographics, and GIS user community.).

As represented by the collared baboons in our study, home range size varied from 1.12 km^2^ to 13.86 km^2^ using the 95% AKDE method (Table 1; Fig 3). Home range estimates using the MCP method ranged from 0.77 km^2^ to 8.83 km^2^. The overall 95% AKDE home range estimates of baboons living in natural environments varied from 6.19 km^2^–13.86 km^2^, compared to 1.31 km^2^–1.79 km^2^ for urban and 1.12 km^2^–2.62 km^2^ for baboon groups living in semi natural environments (Table 1). Home range size differed among behavioral categories, although the difference was not statistically significant (Kruskal–Wallis test: H = 5.60, df = 2, p = 0.061). Descriptively, however, baboons living in natural environments had larger observed home ranges than those in semi-natural and urban environments.

**Table 1:** Home range estimations (95% AKDE and MCP) and minimum daily path lengths of individual GPS-collared hamadryas baboons in Saudi Arabia (August 2022-May 2023) along with other parameters.

Collar ID	Region	No. of days active	No. of fixes	Category	95% AKDE home range (km^2^)	MCP home range (km^2^)	Median of minimum daily path length (km/day)	Range of minimum daily path length (km/day)
N1	Makkah	275	1,641	Natural	8.79	8.83	2.72	0.03-20.60
N2	Al Bahah	275	1,619	Natural	6.19	4.03	3.02	0.05-8.80
N3	Najran	264	1,595	Natural	13.86	8.40	3.93	0.01-13.18
SN1	Al Bahah	282	1,630	Semi-natural	2.62	2.35	4.17	0.01-19.40
SN2	Aseer	256	1,758	Semi-natural	1.18	1.72	2.34	0.14-5.13
SN3	Aseer	283	1,701	Semi-natural	1.12	0.77	1.49	0.2-16.70
U1	Makkah	265	1,548	Urban	1.24	1.03	2.27	0.45-4.2
U2	Jazan	259	1,515	Urban	1.79	1.63	2.13	0.06-4.31
U3	Aseer	168	955	Urban	1.31	0.94	2.02	0.01-4.85

### Daily path lengths

**Fig 4:**
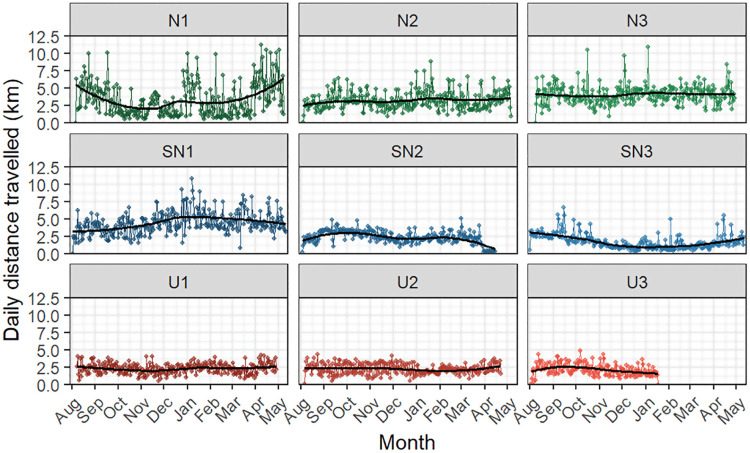
Monthly variation in daily distance travelled by GPS-collared hamadryas baboons (*Papio hamadryas*) in Saudi Arabia (August 2022-May 2023). Each panel represents an individual baboon, with black lines indicating smoothed trends across months.

The mean minimum daily path length across all collared baboons was 2.95 ± 1.8 km/day (median 2.6 km/day; range 0.01–20.6 km) with substantial variation across ecological settings (Fig 4). Natural populations exhibited the longest daily travel distances, followed by semi-natural groups, while urban individuals showed the most restricted movements (Table 1). Among individual collars, SN1, N3, and N1 recorded the greatest daily distances, occasionally exceeding 13–20 km/day, indicating extensive ranging behavior in more natural or semi-natural groups. In contrast, baboons living in urban areas (U1–U3) exhibited short and consistent daily movements of approximately 2–4 km/day (table 1).

A Kruskal–Wallis rank sum test indicated a highly significant effect of category on daily movement distance (χ² = 235.91, df = 2, *p* < 0.0001). Post-hoc pairwise Wilcoxon rank sum tests with Bonferroni correction confirmed that all pairwise comparisons were statistically significant (*p* < 0.001). Specifically, urban individuals travelled significantly shorter daily distances compared to both semi-natural (*p* < 0.0001) and natural groups (*p* < 0.0001), while semi-natural individuals also travelled less than natural ones (*p* < 0.0001).

### Time spent in Urban areas

The proportion of daytime (0600–1800) spent in urban environments varied markedly among baboons from different behavioral categories (Table 2). Urban individuals spent the greatest proportion of daytime around built-up areas (54.96%), followed by semi-natural individuals (40.58%), whereas natural individuals spent minimal time around built-up areas (7.18%). At the individual level, urban baboon U1 showed the strongest urban association, spending over 64% of his daytime within built-up areas. In contrast, natural baboon N1 was largely confined to natural habitats, with less than 2% of his daytime locations recorded around built-up areas. Values tended to differ among Natural, Semi-natural, and Urban groups, although the difference was only marginally significant (Kruskal–Wallis: χ² = 5.96, df = 2, p = 0.05).

**Table 2:** Percentage of daytime GPS fixes (0600-1800) recorded in urban habitats for GPS-collared hamadryas baboons across different study sites in Saudi Arabia (August 2022- May 2023).

Collar ID	Category	Percentage of daytime spent in urban areas	Average (%)
N1	Natural	1.85	7.18
N2	Natural	9.58	
N3	Natural	10.11	
SN1	Semi-natural	23.84	40.58
SN2	Semi-natural	47.28	
SN3	Semi-natural	50.61	
U1	Urban	64.29	54.96
U2	Urban	43.63	
U3	Urban	56.96	

A strong negative relationship was detected between urban space use and home range size (Spearman’s rank correlation: ρ = −0.82, p = 0.011), indicating that baboons spending a greater proportion of daytime in urban areas exhibited smaller home ranges.

## Discussion

GPS tracking of natural, semi-natural, and urban hamadryas baboons revealed marked differences in spatial behavior among these categories. The data clearly show varying degrees of association with human settlements among the troops, highlighting how proximity to people shapes daily activity and space use. Moreover, the findings demonstrate that anthropogenic environments significantly modify movement patterns, home-range size, and spatial stability. Together, these results provide important insights into the behavioral ecology of hamadryas baboons in human-altered landscapes and offer valuable guidance for mitigating human–baboon conflict in Saudi Arabia and similar contexts elsewhere.

Overall, GPS collar performance was robust with high fix success rates ensuring that observed spatial patterns accurately represent natural movement behaviors. Comparable fix success and accuracy have been reported in studies of other medium to large-bodied primates, including baboons, underscoring the reliability of GPS-based behavioral inference [12,45,46].

Empirical variograms revealed clear differences in spatial autocorrelation and movement variability among behavioral categories. Baboons in natural environments exhibited the highest semivariance values, indicating greater spatial displacement and extensive ranging behaviour. Semi-natural individuals showed intermediate semivariance values and more variable temporal structures, suggesting flexible space use. In contrast, urban individuals consistently exhibited the lowest semivariance values, reflecting restricted movement and smaller spatial ranges. These patterns are consistent with movement ecology theory, where animals relying on dispersed natural resources range over larger areas, whereas individuals using predictable anthropogenic resources exhibit reduced movement and spatially constrained ranging behavior [24,47,48].

Marked differences in home-range size of natural and urban categories were observed, consistent with the influence of habitat type and human proximity. Baboons occupying natural areas exhibited larger home ranges than urban-associated baboons, whereas semi-natural groups showed home range sizes closer to urban populations rather than occupying a truly intermediate position. The reason for this may be that access to predictable anthropogenic resources reduces the need for extensive ranging [1,24,49]. Previously, in Saudi Arabia, Boug reported an annual MCP home range of approximately 6.87 km² for a hamadryas baboon troop at the Al Hada site near Taif, which falls within the range observed for natural populations in the present study [30]. In Amboseli, Kenya, yellow baboons (*Papio cynocephalus*) that exploited food from a garbage dump exhibited more regular daily activity, traveled shorter distances, and occupied smaller home ranges compared to nearby un-provisioned groups [49]. Comparable trends have been observed in chacma baboons (*Papio ursinus*) from the Western Cape, South Africa, where a troop with access to human food resources in a resource-rich, modified landscape maintained a much smaller annual range (~9.5 km²) than a similar-sized group inhabiting a less human-influenced area (~16.7 km²) [1].

Our findings align with the RDH-based hypothesis: baboons living in urban environments, which rely on highly clumped and predictable anthropogenic food sources such as refuse dumps and handouts, maintain considerably smaller home ranges. In addition, these anthropogenic foods are often energy-dense and nutritionally concentrated, which may further reduce the need for extensive foraging movements in urban-associated groups [50]. In contrast, baboons living in natural arid habitats, where vegetation and water were sparse and patchily distributed, were required to travel across much larger areas to access dispersed resources. Similar patterns have been documented in other primate species inhabiting fragmented or human-altered habitats [51,52]. These findings extend the RDH framework to a context of rapid urbanization in the Arabian Peninsula, where natural landscapes transition to peri-urban mosaics. However, our RDH-based interpretation should be considered with the limitation that group size was not included as a predictor of home-range size or daily path length. Comparable group-size estimates were not available across all sites, and in hamadryas baboons this is further complicated by their multilevel dynamic social organization, in which OMUs (One male units) are nested within clans, bands, and troops showing fission-fusion frequently. Future studies combining GPS tracking with systematic counts of OMU, clan, and band size would help further disentangle the relative effects of social organization and resource distribution on ranging behavior.

Beyond habitat modification alone, the movement of baboons into urban areas is also driven by predictable anthropogenic food sources including agricultural fields, tourist feeding points, and especially garbage dumps which further intensify their reliance on human-derived resources [6]. Similar anthropogenic gradients have influenced ranging behavior in chimpanzees [47] and macaques [53], suggesting that flexible spatial strategies in response to resource distribution are a common primate adaptation. These results also echo findings from South African baboon populations, where troops feeding in agricultural and urban zones displayed restricted ranges compared to those in protected areas [1,12,24]. Thus, the reduced home range in baboons living in urban environments likely represents a behavioral adaptation to resource predictability rather than an intrinsic ecological preference.

The variation in minimum daily path lengths among baboon groups reflects differing energetic and ecological contexts. Urban individuals’ restricted and consistent movements indicate behavioral adaptation to abundant and predictable anthropogenic foods, while groups living in natural and semi-natural environments maintain broader or more flexible ranging strategies in response to variable resource availability. These trends are consistent with studies of other *Papio* populations showing that access to reliable human-derived foods reduced travel effort and daily range [8,24]. Comparable patterns were reported by Boug at the Al Hada site near Taif, where hamadryas baboons showed daily movements ranging from 1.01 km to 14.03 km, with increased travel distances observed when food provisioning declined due to temporary road blockages [30]. The minimum daily path lengths observed in the present study fall within this range, reinforcing the influence of food availability and human interaction intensity on movement dynamics. It is however important to note that different methodological approaches to calculating daily path length can yield varying estimates [54], and this may further contribute to differences observed across studies. The pattern across categories observed in our study aligns with optimal foraging theory, where predictable, energy-rich resources allow animals to minimize movement costs [55]. In contrast, natural groups in arid or seasonally variable landscapes expand their foraging ranges when resources are scarce [9,56,57].

The proportion of daytime locations in built-up (urban) areas varied among behavioral categories, although marginally significant statistically. Urban groups appear to be highly habituated to human environments, reflecting a strong behavioral adaptation to anthropogenic resources spending a large amount of time in urban environments. Historical reports similarly documented that by the 1980s, some troops near cities derived nearly half of their diet from refuse dumps and spent most of their time around these sites, traveling only short distances [6,58].

These anthropogenic associations have important ecological and conflict implications. Access to calorie-rich human food wastes may alter body condition and energy balance, potentially accelerating reproductive rates or reducing inter-birth intervals thus having long term demographic consequences [59]. In Saudi Arabia, urban-edge baboon populations have shown a high prevalence of parasitic and bacterial infections, underscoring the physiological costs of proximity to human settlements [60–63]. These studies also highlight concerns regarding zoonotic transmission, as several of the identified pathogens are shared between baboons and humans. Moreover, altered movement patterns and concentrated resources in urban settings can disrupt traditional social structures of baboons, weakening dominance hierarchies and affiliative bonds that are closely linked to female reproductive success and survival [64]. Reliance of baboons living in urban areas on predictable human foods (e.g., waste, crops, intentional feeding) reduces their need to range far and stabilizes their movements, but it also brings them into frequent contact with people [9]. In Cape Town, for example, baboons strongly prefer urban and suburban habitats that offer proximate food and shelter [1], which has produced intense competition with humans. Similarly, in southwest Saudi Arabia rapid urban growth has attracted baboons to cities; their habituation has led to serious problems such as aggressive encounters, property damage, and public health risks [6,58]. Indeed, Angelici *et al*. [6] note that high levels of human–baboon overlap in urban areas are linked to nuisance behaviors and even zoonotic disease transmission, while for baboons, such proximity increases exposure to risk of injury or culling and has adverse impact on physiology and behavior [8,65]. In line with these observations, our findings show that baboons adapted to natural environments remain largely restricted to wilderness, whereas baboons living in urban areas frequently utilize human-dominated areas, a pattern that may elevate the potential for human–baboon conflict and zoonotic transmission. In an evolutionary-ecological sense, anthropogenic resource predictability may lower the cost of living in larger groups or at high density, but it effectively creates a big subpopulation of baboons at the human–wildlife interface [1].

## Conclusion

Our results show that baboons spending a greater proportion of time in urban areas tended to have smaller home ranges and shorter daily movements, whereas individuals using predominantly natural environments ranged more widely. Our results quantitatively describe differences in space use among baboon groups occupying natural, semi-natural, and urban environments, providing a framework that may help managers tailor interventions to different conflict contexts. From a management perspective, interventions should be matched to category: groups living in natural environments are best managed with habitat protection, semi-natural groups warrant targeted population management coupled with conflict mitigation, while urban groups require a more intensive integrated approach. Since, urban-associated baboons concentrated their movements within relatively restricted areas, management should prioritize reducing access to predictable anthropogenic food sources through secure waste systems, improved refuse management, and public education to discourage feeding. (cf. [6,24]). Spatial planning measures, including buffer zones around settlements, waste-disposal areas, tourist sites, and key baboon ranging areas, may also help reduce repeated human–baboon encounters. Restoring and maintaining natural foraging habitat will also help meet energetic requirements away from settlements. In future, integrating high-resolution GPS telemetry with systematic behavioral observation and expanding drone and computer-vision-based monitoring will strengthen adaptive, evidence-based conservation planning. Together, these approaches offer a pragmatic pathway toward sustainable human–baboon coexistence while minimizing conflict and preserving ecological function in Saudi Arabia.
